# *Fusarium graminearum* ATP-Binding Cassette Transporter Gene *FgABCC9* Is Required for Its Transportation of Salicylic Acid, Fungicide Resistance, Mycelial Growth and Pathogenicity towards Wheat

**DOI:** 10.3390/ijms19082351

**Published:** 2018-08-10

**Authors:** Peng-Fei Qi, Ya-Zhou Zhang, Cai-Hong Liu, Jing Zhu, Qing Chen, Zhen-Ru Guo, Yan Wang, Bin-Jie Xu, Ting Zheng, Yun-Feng Jiang, Jiang-Ping Wang, Cai-Yi Zhou, Xiang Feng, Li Kong, Xiu-Jin Lan, Qian-Tao Jiang, Yu-Ming Wei, You-Liang Zheng

**Affiliations:** Triticeae Research Institute, Sichuan Agricultural University, Chengdu 611130, China; 15828521149@163.com (Y.-Z.Z.); 18280184730@163.com (C.-H.L.); sicauZJing@163.com (J.Z.); qingchen83@sicau.edu.cn (Q.C.); guozhenru@stu.sicau.edu.cn (Z.-R.G.); wyan810@163.com (Y.W.); binjiexu@outlook.com (B.-J.X.); TingZheng521@hotmail.com (T.Z.); jiangyunfeng2018@163.com (Y.-F.J.); jiangpingwang@stu.sicau.edu.cn (J.-P.W.); zhoucaiyi@stu.sicau.edu.cn (C.-Y.Z.); fengxiang@stu.sicau.edu.cn (X.F.); kongli088@163.com (L.K.); lanxiujin@163.com (X.-J.L.); qiantaojiang@sicau.edu.cn (Q.-T.J.); ylzheng@sicau.edu.cn (Y.-L.Z.)

**Keywords:** ABC transporter, SA, Fusarium head blight, mycotoxin

## Abstract

ATP-binding cassette (ABC) transporters hydrolyze ATP to transport a wide range of substrates. *Fusarium graminearum* is a major causal agent of Fusarium head blight, which is a severe disease in wheat worldwide. *FgABCC9* (*FG05_07325*) encodes an ABC-C (ABC transporter family C) transporter in *F. graminearum*, which was highly expressed during the infection in wheat and was up-regulated by the plant defense hormone salicylic acid (SA) and the fungicide tebuconazole. The predicted tertiary structure of the FgABCC9 protein was consistent with the schematic of the ABC exporter. Deletion of *FgABCC9* resulted in decreased mycelial growth, increased sensitivity to SA and tebuconazole, reduced accumulation of deoxynivalenol (DON), and less pathogenicity towards wheat. Re-introduction of a functional *FgABCC9* gene into Δ*FgABCC9* recovered the phenotypes of the wild type strain. Transgenic expression of *FgABCC9* in *Arabidopsis thaliana* increased the accumulation of SA in its leaves without activating SA signaling, which suggests that *FgABCC9* functions as an SA exporter. Taken together, *FgABCC9* encodes an ABC exporter, which is critical for fungal exportation of SA, response to tebuconazole, mycelial growth, and pathogenicity towards wheat.

## 1. Introduction

Fusarium head blight (FHB) is a severe disease of wheat in the world, which is mainly caused by the ascomycete fungus *Fusarium graminearum* [1,2,3]. FHB causes yield loss and contamination of seeds by the accumulation of trichothecene mycotoxins (mainly deoxynivalenol [DON]), which threatens food and feed security [4,5]. FHB has not been well-controlled due to the complexity of wheat resistance to *F. graminearum*. Therefore, it is necessary to understand the molecular mechanism of interaction between *F. graminearum* and wheat, which would be valuable for designing a proper strategy for FHB disease management.

ATP-binding cassette (ABC) transporters hydrolyze ATP to transport a wide range of substrates and can be broadly categorized as importers or exporters in the cell membrane depending on the direction of transportation relative to the cytoplasm [6,7]. Members of this superfamily have conserved domain architecture and have been divided into seven families (A–G) in fungi, plants, and animals [8,9,10]. Recent studies demonstrated that the ABC transporter family C (ABC-C) as a cell membrane protein is important for fungal pathogens in response to fungicide [11,12,13].

In the genome of *F. graminearum*, there are 16 genes belonging to ABC-C [9]. Many of them are expressed at much higher or lower levels during the infection process when compared with their expression in an axenic culture [14,15,16]. *FgABCC15* (*FG05_10995*) played important and diverse roles in both fungicide resistance and pathogenesis of *F. graminearum* [9,15]. Considering the importance of ABC-C transporters and the hazards of FHB, it is still necessary to characterize other ABC-C transporters in *F. graminearum*.

Salicylic acid (SA) is a critical plant defense hormone, which contributes to plant defense against a wide range of pathogens with biotrophic and hemibiotrophic lifestyles [17,18,19]. SA triggers systemic acquired resistance (SAR) in plants and upregulates the expression of a set of genes encoding pathogenesis-related (PR) proteins in tobacco and *Arabidopsis thaliana* [20,21].

The contribution of SA to the wheat/*F. graminearum* interaction remains unclear. Infection of wheat spikes with *F. graminearum* resulted in a significant increase in the accumulation of SA [22], which suggests that SA is important in wheat defense against *F. graminearum*. Over-expression of Nonexpresser of PR Genes 1 (*NPR1*) of *A. thaliana* enhanced the FHB resistance level in transgenic wheat [23]. SA has a significant and direct impact on *F. graminearum* by reducing the efficiency of germination and growth [24]. Nevertheless, exogenous application of SA in wheat spikes cannot increase its resistance against FHB as expected, which possibly resulted from the capacity of *F. graminearum* to metabolize and export SA [24]. However, the molecular mechanism on the exportation of SA in *F. graminearum* is still not well understood.

In this paper, we characterized an ABC-C transporter gene (*FG05_07325*) in *F. graminearum*. It was named *FgABCC9* following Kovalchuk and Driessen [9]. *FgABCC9* was highly expressed during the infection of wheat and was upregulated by SA and by the fungicide tebuconazole [12,22,25]. The objective of this research was to understand the mechanism for the exportation of SA in *F. graminearum* by analyzing the function of *FgABCC9* and elevating its effect on fungal mycelial growth and pathogenicity towards wheat. This research would be helpful for understanding the contribution of SA to the wheat/*F. graminearum* interaction.

## 2. Results

### 2.1. Sequence Analysis

**Figure A1:**
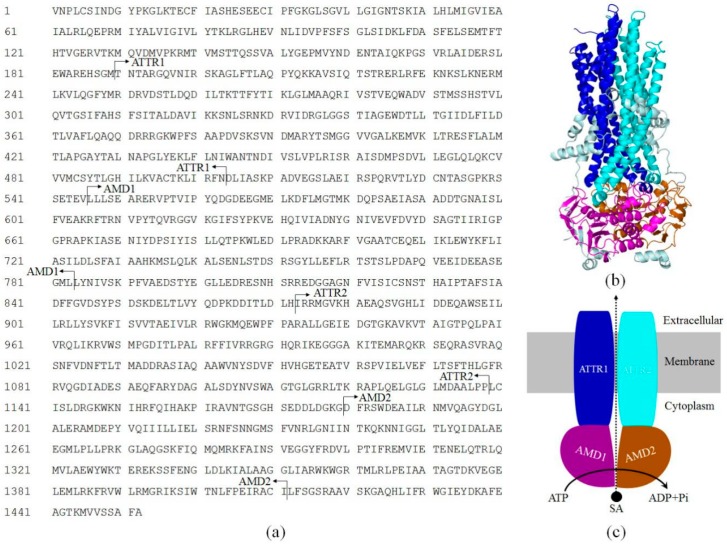
Structure of FgABCC9. (**a**) Distribution of conserved protein domain in the amino acid sequence of FgABCC9. (**b**) Predicted tertiary structure of FgABCC9 protein. Dark blue, purple, cyan, and brown colors indicate the “ATTR1” (ABC transporter transmembrane region 1; pfam00116; 190-503 aa), “AMD1” (ABC transporter C family MRP (multidrug resistance-associated protein) domain 1; cd03250; 546-783 aa), “ATTR2” (ABC transporter transmembrane region 2; pfam00664; 873-1138 aa), and “AMD2” (ABC transporter C family MRP domain 2; cd03250; 1180-1411 aa), respectively. (**c**) Schematic of ABC exporters [26].

*FgABCC9* gene is 4533 bp in length with 3 exons and 2 introns and its open reading frame is 4359 bp. Its deduced amino acid sequence (Appendix Figure A1a) includes the ABC transporter transmembrane region 1 (ATTR1, pfam00116, 190-503 aa), the ABC transporter C family MRP domain 1 (AMD1, cd03250, 546-783 aa), the ABC transporter transmembrane region 2 (ATTR2, pfam00664, 873-1138 aa), and the ABC transporter C family MRP domain 2 (AMD2, cd03250, 1180-1411 aa). The predicted tertiary structure of FgABCC9 was shown in Appendix Figure A1b, which is consistent with the model structure (Appendix Figure A1c) for ABC exporters [26] indicating that *FgABCC9* encodes an ABC exporter.

### 2.2. Deletion and Complementation of FgABCC9 in F. graminearum

**Figure 1:**
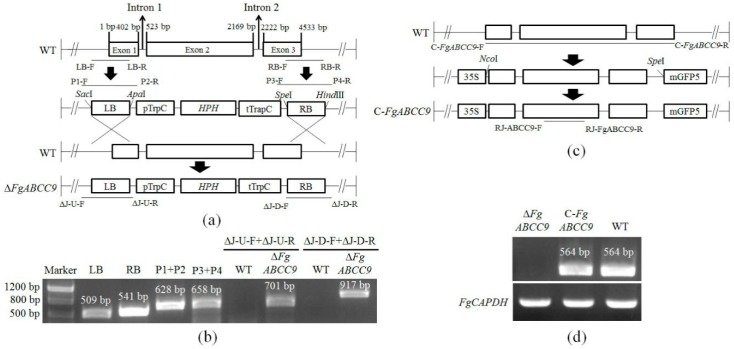
Construction of deletion and complementation mutants for *FgABCC9*. (**a**) The left border (LB) and the right border (RB) were amplified from the wild type (WT) strain to construct the recombinant plasmid. Δ*FgABCC9* mutants were then created by homologous recombination between the plasmid and *FgABCC9*. (**b**) PCR verification of Δ*FgABCC9*. (**c**) Gene sequence of *FgABCC9* was amplified from the genomic DNA of WT and ligated into the complementation vector. The T-DNA region of the complementation vector was inserted into the genome of Δ*FgABCC9* to create C*-FgABCC9*. *Sac*I, *Apa*I, *Spe*I, *Hind*III, *Nco*I, and *Spe*I show the restriction enzymes used. (**d**) RT (reverse transcription)-PCR of *FgABCC9* in C*-FgABCC9* using RJ-*ABCC9*-F + RJ-*ABCC9*-R. *FgGAPDH* was used as reference. The black lines under the elements in (**a**) and (**c**) represent the targeted location of primers listed in Table 1. LB-F + LB-R, RB-F + RB-R, C-*FgABCC9*-F + C-*FgABCC9*-R were located in the genomic sequence of *FgABCC9* of the WT strain. P1-F + P2-R and P3-F + P4-R were mapped in the T-DNA fragment of the pRF-HU2 vector. ΔJ-U-F, ΔJ-U-R, ΔJ-D-F, and ΔJ-D-R were, respectively, positioned into the upstream and downstream of inserted T-DNA sequence of Δ*FgABCC9*. All the PCR products were verified by sequencing in the commercial company (Qingke, Chengdu, China).

To disrupt the function of *FgABCC9* in *F. graminearum*, the two flanking regions of *FgABCC9* were amplified from the genomic DNA of *F. graminearum* by using P1-F + P2-R and P3-F + P4-R, respectively (Figure 1a), which were then inserted into the pRF-HU2 vector (Figure 1a). Deletion mutants (Δ*FgABCC9*) were created by replacing the entire *FgABCC9* gene with *HPH*, which is known as a fungal selectable marker gene, using a targeted gene replacement strategy through homologous recombination.

To ensure that the construct was integrated at the intended homologous site (Figure 1b), the primer pairs ΔJ-U-F + ΔJ-U-R and ΔJ-D-F + ΔJ-D-F were used to test Δ*FgABCC9*, which is shown in Figure 1a. The two primer pairs provided expected PCR bands in Δ*FgABCC9* (Figure 1b), which were confirmed by sequencing. These results show that *FgABCC9* was successfully removed from the genome of Δ*FgABCC9*.

To create complementation mutants (C-*FgABCC9*), a fragment containing the promoter and open reading frame of *FgABCC9* was introduced into Δ*FgABCC9* (Figure 1c). Reverse transcription PCR (RT-PCR) showed that the *FgABCC9* gene was normally expressed in C-*FgABCC9* as in WT and no expression was observed in Δ*FgABCC9* (Figure 1d).

### 2.3. Importance of FgABCC9 for Mycelial Growth and Fungal Response to Stress Conditions

**Figure 2:**
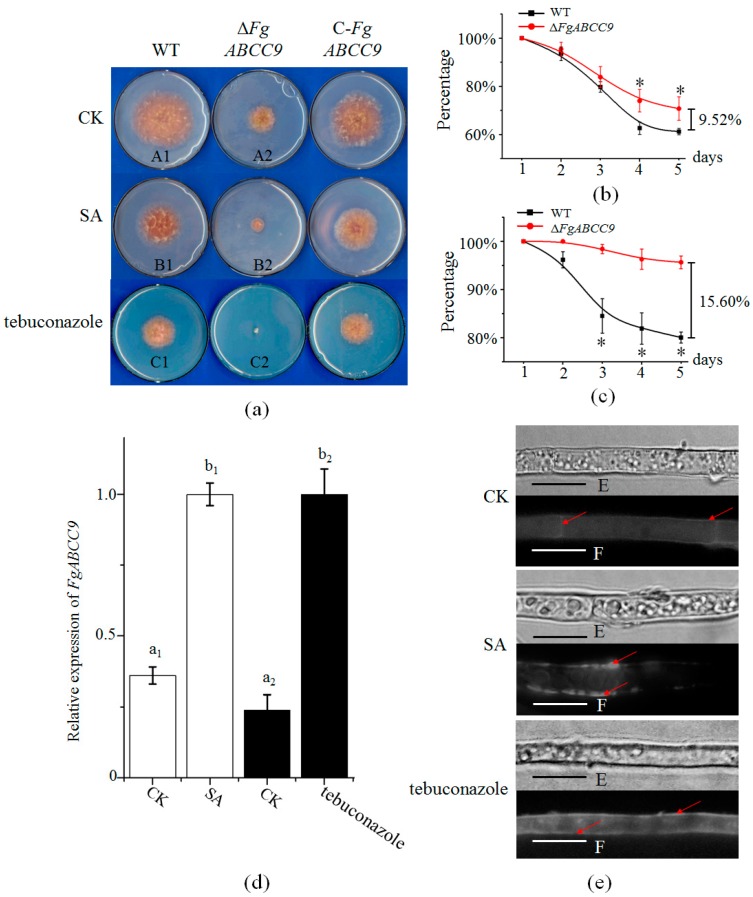
Effect of *FgABCC9* on mycelial growth. (**a**) Mycelial growth on mSNA (modified Synthetischer Nährstoffarmer Agar) plates with SA and tebuconazole. CK, control treatment. Plates were photographed on the fourth day post inoculation. (**b**) Percentage of mycelial growth inhibited by SA ([A1-B1]/A1 for WT, [A2-B2]/A2 for Δ*FgABCC9*). (**c**) Percentage of mycelial growth inhibited by tebuconazole ([A1-C1]/A1 for WT, [A2-C2]/A2 for Δ*FgABCC9*). Asterisks represent significance at *p* < 0.05. A1, B1, and C1 indicate mycelial areas of WT strain under the CK, SA, and tebuconazole treatments, respectively. A2, B2, and C2 indicate mycelial areas of Δ*FgABCC9* under the CK, SA, and tebuconazole treatments, respectively. The experiments were repeated three times with 10 plates for each treatment. (**d**) Expression changes of *FgABCC9* under SA and tebuconazole treatments in the WT strain. Different letters above each column indicate significance at *p* < 0.05. (**e**) Subcellular localization of the FgABCC9 protein. The fluorescent signal is marked with red arrows. E, Optical microscope. F, fluorescence microscope. Scale bar, 10 µm.

Mycelial growth of WT (wild type), Δ*FgABCC9*, and C-*FgABCC9* strains was compared on mSNA (modified Synthetischer Nährstoffarmer Agar) plates (Figure 2a). Δ*FgABCC9* grew much slower than WT and C-*FgABCC9*. The expression of *FgABCC9* could be induced by the plant defense hormone SA and the fungicide tebuconazole (Figure 2d), which was previously reported [12,24]. To clarify whether *FgABCC9* was involved in mechanisms alleviating SA and tebuconazole, mycelial growth of WT, Δ*FgABCC9* and C-*FgABCC9* were elevated on mSNA plates supplemented with 0.9 mM SA and 0.5 mM tebuconazole, respectively (Figure 2a). When compared to WT, Δ*FgABCC9* was more sensitive to SA and tebuconazole, which reveals the important role of *FgABCC9* in a fungal response to SA and tebuconazole (Figure 2b,c).

The FgABCC9 protein was usually distributed in the septa zone and the cell membrane (Figure 2e). Consistent with its expression at the RNA level, the accumulation of the FgABCC9 protein in the fungal cell membrane was enhanced when treated with SA and tebuconazole (Figure 2e), which further demonstrates the key role of FgABCC9 in the transportation of SA and tebuconazole in *F. graminearum*.

### 2.4. FgABCC9 Affects Pathogenicity and DON Production

**Figure 3:**
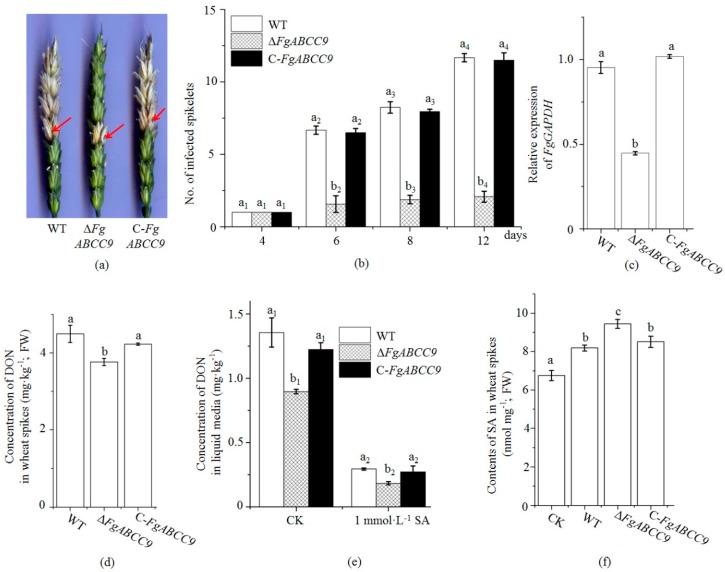
*FgABCC9* affected pathogenesis of *F. graminearium* in spikes. (**a**) FHB disease in wheat heads photographed on the eighth day after inoculation. Arrows indicate the inoculated spikelets. (**b**) the numbers of infected and bleached spikelets on the fourth, sixth, eighth, and twelfth day after inoculation. (**c**) Relative expression level of *FgGAPDH (*glyceraldehyde 3-phosphate dehydrogenase gene of *F. graminearium*, *FG05_06257*) in wheat spikes inoculated with WT, Δ*FgABCC9*, and C-*FgABCC9*, respectively. (**d**) DON contents in wheat spikes. (**e**) Concentration of DON in liquid medium. (**f**) Comparison of the accumulation of SA in wheat spikes after inoculation. Values are the mean ± standard deviation. Different letters above each column indicate a significant difference (*p* < 0.05). FW, fresh weight.

To clarify whether *FgABCC9* affected pathogenicity of the *F. graminearum*, two fully developed florets of a central spikelet were point-inoculated with conidial suspensions of WT, Δ*FgABCC9*, and C-*FgABCC9*, respectively. In agreement with its slower mycelial growth on plates and increased sensitivity to SA (Figure 2a), spikes inoculated with Δ*FgABCC9* show much less visual disease symptom and a lower fungal biomass when compared with those inoculated with WT and C-*FgABCC9* (Figure 3a–c)*.* The concentrations of DON in the liquid culture and in wheat spikes were compared as well. Δ*FgABCC9* had a much lower DON production than WT and C-*FgABCC9* (Figure 3d,e).

Considering that *FgABCC9* encodes an SA exporter, we compared the contents of SA in spikes inoculated with WT and Δ*FgABCC9*. Spikes infected with Δ*FgABCC9* accumulated a higher concentration of SA (Figure 3f), which suggests that the export of SA by FgABCC9 is critical for the balance between the degradation of wheat endogenous SA by *F. graminearum* and the toxicity of SA to *F. graminearum* (reduction in mycelial growth and conidial germination [24]) during the infection in wheat.

### 2.5. Evaluation of the Function of FgABCC9 in A. thaliana

**Figure 4:**
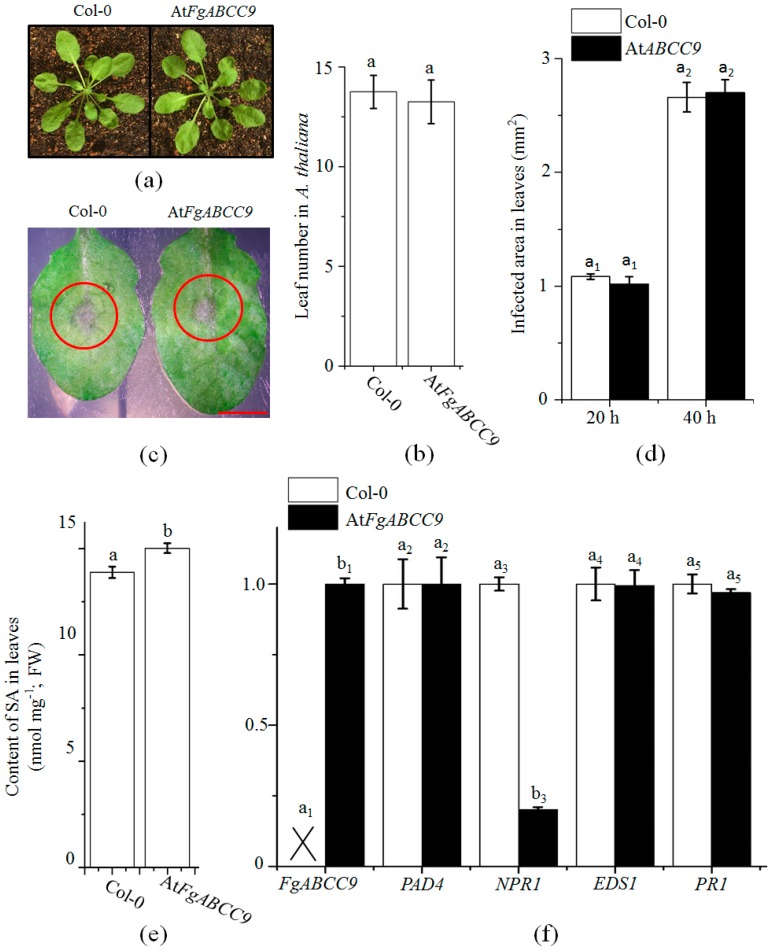
Evaluation of the function of *FgABCC9* in *A. thaliana*. (**a**) The WT and At*FgABCC9* plants on the 30th day under SD condition. (**b**) Comparison of leaf numbers of WT and At*FgABCC9* plants as used in (**a**). (**c**) Comparison of visual disease symptoms in leaves of WT and At*FgABCC9* plants infected by *F. graminearium* at the 48th hour after inoculation. Scale bar, 1 cm (**d**) Comparison of infected areas in leaves at the 20th and 40th hour after inoculation. (**e**) Comparison of the contents of SA in leaves (without inoculation) of WT and At*FgABCC9* plants on the 30th day post germination under the SD (short day) condition. (**f**) Comparison of gene expression in leaves (without inoculation) of WT and At*FgABCC9* plants by qPCR. The cross indicates the absence of *FgABCC9* from Col-0. Different letters above each column indicate significance at *p* < 0.05.

To confirm its function, *FgABCC9* was expressed in *A. thaliana*. The leaves of At*FgABCC9* accumulated more SA than those of Col-0 (Figure 4e) even though there was no visual difference between Col-0 and At*FgABCC9* plants (Figure 4a,b). To make sure the increased accumulation of SA affected expression of SA-related genes, we compared the expression of *NPR1*, *EDS1*, *PAD4*, and *PR1* in the leaves of transgenic and WT plants (Figure 4f). *NPR1* is a master regulator of SA-mediated transcriptional reprogramming and immunity and it functions as a transcriptional coactivator [27]. *EDS1* and *PAD4* as part of a central regulator complex contribute to the positive feedback loop of SA accumulation [28]. *PR1* is one of the genes that is induced by SA and often used as the marker for SA signaling [29]. Unexpectedly, only the expression of *NPR1* was affected, which indicated that transgenic expression of *FgABCC9* did not activate SA signaling.

To clarify whether *FgABCC9* affected *A. thaliana* resistance against *F. graminearum*, the leaves of At*FgABCC9* and Col-0 were point-inoculated with conidial suspension of the WT strain. Consistent with the qPCR data in Figure 4f, there was no significant difference between the resistance levels of At*FgABCC9* and Col-0 (Figure 4c,d).

## 3. Discussion

The superfamily of the ABC transporter is one of the largest gene families in fungi. It is involved in the transport of a broad range of substrates across biological membranes. ABC transporters, particularly those with a multidrug resistance (MDR) domain or a pleiotropic drug resistance (PDR) domain, are important in the resistance of fungal pathogens to xenobiotics [11]. *F. graminearum*, which is an ascomycete fungus, is a major causal pathogen for FHB disease in wheat. *F. graminearum* has 62 ABC transporter genes, which were divided into seven families (A–G) according to domain architecture and phylogenetic data [9]. *FgABCG6* (*FG05_04580*) with a PDR domain plays a role in protecting the fungus from antifungal compounds and helps combat unidentified wheat defense compounds during disease development [30]. *FgABCC15* (*FG05_10995*) with an MRP domain plays important and diverse roles in both fungicide resistance and pathogenesis of *F. graminearum* [15]. In this study, we characterized a new ABC-C transporter gene with an MRP domain (*FgABCC9*; *FG05_07325*). Our data demonstrated that FgABCC9 was an ABC exporter, which was important for the export of SA by *F. graminearum* under SA stress and for fungal response to tebuconazole and pathogenesis in wheat. Our results together with the previous reports suggested that manipulation of the expression level or activity of ABC transporters would be a hopeful way to manage FHB disease.

SA is one of the key plant defense hormones, which is associated with resistance against *F. graminearum* in wheat [22,23,24]. Nevertheless, the contribution of SA to wheat/*F. graminearum* interaction remains unclear. SA has a significant and direct impact on *F. graminearum* since it reduces the efficiency of germination and mycelial growth at higher concentrations [24] by down regulating the chitin synthase gene *FgCHS8* and the cis-12 linoleic acid isomerase gene *FgLAI12* [31,32]. Wheat spikes accumulate more SA when infected with *F. graminearum* [22], which suggests an important role of SA in wheat FHB resistance. Unexpectedly, exogenous application of SA cannot improve the wheat resistance level against FHB possibly due to the fact that *F. graminearum* can reduce the toxicity of SA by its capacity to metabolize and export SA [24]. This research showed the importance of FgABCC9 as an SA exporter for *F. graminearum* under SA stress and during infection in wheat. Deletion of the *FgABCC9* gene resulted in enhanced fungal sensitivity to SA (Figure 2a), increased accumulation of SA (Figure 3f), lower fungal biomass (Figure 3c), and less disease symptom (Figure 3a,b) in wheat spikes after inoculation. The presence of FgABCC9 was critical for fungal degradation of SA under SA stress (Figure 2a). Therefore, we speculated that Δ*FgABCC9* could not efficiently degrade SA in wheat spikes as the WT strain, which results in enhanced accumulation of SA in spikes inoculated with Δ*FgABCC9* (Figure 3f). It also suggested that *F. graminearum* reduced the accumulation of SA in spikes when FgABCC9 functioned normally and made sure that the concentration of endogenous SA was below the toxicity threshold. Δ*FgABCC9* might not completely lose the ability to export SA from the cytoplasm to the extracellular space since it is hard to identify all the SA exporters in one experiment. However, even the partial reduction of this ability led to lower fungal biomass and less disease symptom, which indicated the contribution of the SA exporter to deal with SA during the infection of *F. graminearum*. These results demonstrated that SA did contribute to wheat resistance against *F. graminearum*.

Transgenic expression of the *FgABCC9* gene did not activate the expression of the *PR1* that is the marker gene for SA signaling (Figure 4f), even though it increased the concentration of SA in the leaves of *A. thaliana* (Figure 4e). Considering that FgABCC9 functions as an SA exporter, we speculated that the content of SA within cytoplasm would not increase.

Tebuconazole is a sterol demethylation inhibitor and is effective in controlling FHB in agricultural production [33,34]. Recent studies show that the 14-α-demethylase encoded by the *Cyp51* gene as a target enzyme affected fungal cell wall sensitivity to tebuconazole. ABC transporters used as efflux pumps were involved in the transportation of tebuconazole in fungi [35,36]. In this study, the expression of the *FgABCC9* gene was upregulated by tebuconazole (Figure 2d,e) and deletion of the *FgABCC9* gene led to increased sensitivity to tebuconazole on mSNA plates (Figure 2a,c). These results suggest that the *FgABCC9* gene is involved in mechanisms alleviating to tebuconazole and that FgABCC9 is an exporter for the fungicide tebuconazole in *F. graminearum* as well.

FHB threatens food and feed security by contaminating seeds with trichothecene mycotoxins. We previously showed that SA significantly inhibited DON production [24] and SA also significantly upregulated the expression level of *FgABCC9* (Figure 2d). In the present study, disruption of the *FgABCC9* gene in *F. graminearum* resulted in reduced accumulation of DON production in wheat spikes and in liquid media (Figure 3d,e). However, the reduced DON contents in spikes inoculated with Δ*FgABCC9* was possibly due to the less fungal biomass (Figure 3c). In the liquid media, the same amount of mycelia (0.2 mg) was used, which demonstrates that the expression of the *FgABCC9* gene was positively associated with DON production in *F. graminearum*. It seems likely that the *FgABCC9* may not be a regulatory target for SA to downregulate DON production in *F. graminearum*.

Members in the ABC-C subfamily have been recognized as export pumps for amphiphilic anions especially for conjugates of lipophilic compounds with glutathione or several other non-toxic anionic residues in *Saccharomyces cerevisiae* [10,37,38,39]. Δ*FgABCC9* showed slower mycelial growth on mSNA plates than WT and C-*FgABCC9* (Figure 2a), which suggested that FgABCC9 is also an exporter for other materials that are essential for normal growth of *F. graminearum*.

## 4. Materials and Methods

### 4.1. Materials

A virulent isolate of *F. graminearum* (DAOM180378) was used for the fungal experiment throughout the paper. *Triticum aestivum* cv. “Roblin” was used for wheat inoculation, which is susceptible to FHB disease. Wheat plants were grown in green houses under 16/8 h day/night cycles at 23/18 °C. Plants were watered as needed and fertilized before sowing with 15-15-15 (N-P-K). Unless specifically noted, all chemicals were purchased from Sigma-Aldrich (St. Louis, MO, USA).

*A. thaliana* ecotype Col-0 was used. Seeds were sown on Murashige and Skoog (MS) medium with 0.7% (weight/volume) agar and were kept at 4 °C for 3 d before being moved to a growth chamber. The WT and transgenic plants were grown side-by-side in the same tray to minimize possible variation of growth conditions. Plants were first grown at 22 °C under a short day (SD) condition for 30 d with a 10 h light/14 h dark photoperiod for an extended vegetative growth phase and were then moved to a long day (LD) (16 h light/8 h dark) condition for reproductive growth. The pCAMBIA1302 vector carrying *FgABCC9* was frozen and then thawed into the *Agrobacterium tumefaciens* strain AGL-1 (Tiangen, Beijing, China). It was transformed into the Col-0 through the floral dip method [40]. Six resistant T_1_ transgenic lines were selected in 50 μg·mL^−1^ hygromycin (HPH) and verified by using PCR. Phenotypic analyses were performed in the T_2_ generation and confirmed in the T_3_ generation. Homozygous plants were used in all experiments.

### 4.2. Sequence Analysis

Nucleotide sequence of *FgABCC9* (*FG05_07325*) was downloaded from the Ensembl fungi database (Available at: http://fungi.ensembl.org/index.html). Primer Premier 5.0 (Premier Biosoft, Palo Alto, CA, USA) software was used to design PCR primers (Table 1). The analysis of sequence similarity and identification of conserved domains were performed using BLASTp on NCBI (Available at: http://blast.ncbi.nlm.nih.gov/Blast.cgi) (Figure 1a). The 3D-protein structure was predicted by Phyre2 [41] and made by PyMol 1.8 (Figure 1b).

### 4.3. Construction of Deletion and Complementation Mutants

Fresh mycelia cultured on mSNA (1 g KH_2_PO_4_, 1 g KNO_3_, 0.5 g MgSO_4_, 0.5 g KCl, 1 g glucose, 1 g sucrose and 20 g agar per liter) plates [24] were used to extract the genomic DNA of *F. graminearum* by using a CTAB method [45]. Disruption of the *FgABCC9* gene in *F. graminearum* was performed, which is shown in Figure 1a. The pRF-HU2 vector [46] was used to create Δ*FgABCC9* mutants through homologous recombination. Transformation of *F. graminearum* was carried out similarly to our previous study [31].

To restore its function, the genomic sequence of *FgABCC9* was amplified by primer pair C-*FgABCC9*-F + C-*FgABCC9*-R, ligated into pCAMBIA1302 vector, and transformed into Δ*FgABCC9* to create C-*FgABCC9* mutants. All the C-*FgABCC9* mutants were verified by RT (reverse transcription)-PCR by using the primer pair RJ-Fg*ABCC9*-F + RJ-*FgABCC9*-CJ-R (Table 1).

### 4.4. Conidial and Mycelial Growth Conditions

Conidia were produced in CMC (carboxymethyl cellulose) medium at 28 °C by shaking (180 rpm) for 5 d [47]. The concentration of conidia was determined with a hemocytometer by microscopy.

The effects of SA and tebuconazole on mycelial growth were tested on mSNA plates. SA (0.9 mM SA) and tebuconazole (0.5 mM) were supplemented after autoclaving. A total of 1000 conidia were inoculated on each mSNA plate and the plates were maintained at 28 °C in darkness. Ten replicates were done for each treatment. The growing mycelia on mSNA plates were scanned by using the EPSON Perfection V700 Photo (Seiko Epson, Bekasi, Indonesia) on day 5 after inoculation. Thereafter, the area of mycelia was measured by Computer Aided Design (CAD) software (version 2007). The B-spline curve for mycelia growth was calculated as Fang et al. [48].

### 4.5. Virulence Assay

To determine the effect of *FgABCC9* on the pathogenicity of *F. graminearum* in wheat heads, two flowering florets of a central spikelet of one head were each inoculated with 1 × 10^3^ conidia. The inoculated heads were sprayed with water and enclosed with a plastic wrap for 48 h at 25 °C. Wheat plants were placed in a controlled-environment room at 25 °C. FHB symptoms were assessed 4 to 12 d after inoculation. Ten plants were used per treatment.

To examine whether *FgABCC9* affected pathogenicity of *F. graminearum* in leaves of *A. thaliana*, 1 × 10^3^ conidia was point inoculated in the middle of leaves wounded by brush. The inoculated leaves were maintained on MS medium at 25 °C for 48 h. Subsequently, the inoculated leaves were scanned by using the EPSON Perfection V700 Photo and ground to powder in liquid nitrogen.

To test whether *FgABCC9* was related to the production of mycotoxin DON in liquid media, a two-stage protocol from References [24,49] was used. The concentration of DON was measured by using the DON ELISA kit (Beacon, Saco, ME, USA) and the Multiskan Spectrum (Thermo Scientific, Vantaa, Finland).

To test whether *FgABCC9* affected the production of DON in wheat spikes, two florets of fully developed spikelets at mid-anthesis were each inoculated with 1 × 10^3^ conidia. After inoculation, the wheat plants were placed in a room as described above. The inoculated spikelets were harvested at the 48^th^ hour (for the following gene expression analysis) and on day 8 after inoculation (for DON analysis). The spikelets were ground into a fine powder in liquid nitrogen. DON was extracted from 100 mg spikelet powder with one mL sterile water at 4 °C for 12 h. Three biological replicates were conducted with at least ten heads per treatment.

### 4.6. Gene Expression Analysis

Total RNA was extracted from fresh powders of mycelia, wheat spikelets, and *A. thaliana* leaves by using the E.Z.N.A.^®^ Total RNA Kit I (Omega Bio-Tek, Norcross, GA, USA) in accordance with the manufacturer’s instructions. RNA was reverse transcribed using the PrimeScript™ RT Reagent Kit with genomic DNA Eraser (Takara, Dalian, China) following the manufacturer’s protocol.

The primer pair Rj-*FgABCC9*-F + Rj-*FgABCC9*-R was used to measure the expression level of *FgABCC9* in *F. graminearum* and *A. thaliana*. Gene expression of *PR1*, *EDS1*, *PAD4*, and *NPR1* was detected in the leaves of *A. thaliana* by using *PR1*-F + *PR1*-R, *EDS1*-F + *EDS1*-R, *PAD4*-F + *PAD4*-R, and *NPR1*-F + *NPR1*-R, respectively (Table 1). The *FgGAPDH* (*FG05_06257*), β-tubulin (*FG05_09530*) and elongation factor 1 (*FG05_08811*) genes were used as reference genes when performing qPCR for the samples of *F. graminearum* [24]. The relative amount of *F. graminearum* was estimated by measuring the expression level of *FgGAPDH* in wheat spike tissue by using qPCR with normalization to three wheat reference genes (*w-GAPDH* (glyceraldehyde-3-phosphate dehydrogenase gene in wheat), NCBI UniGene Ta.66461; *Aox* (aldehyde oxidase gene), Ta.6172; *hn-RNP-Q* (heterogeneous nuclear ribonucleoprotein Q gene), Ta.10105) [24]. The *ACT2*, *UBQ10*, and *EF-1α* genes [42] were used as reference genes when doing qPCR in the samples of *A. thaliana*. qPCR was performed by a MyiQ Real-Time PCR Detection System (Bio-Rad, Hercules, CA, USA). All of the primers mentioned above were listed in Table 1.

### 4.7. Quantification of SA

The wheat spikelet and *A. thaliana* leaf samples for qPCR analysis were utilized for the quantification of SA as well. To prepare samples of *A. thaliana*, leaves of WT and transgenic plants grown under the SD condition without inoculation were harvested and ground to a fine powder in liquid nitrogen, respectively. Three biological replicates were conducted with at least six plants per treatment. Quantification of SA was performed as described previously [50].

### 4.8. Microscopic Assay

The C-*FgABCC9* strains harboring a green fluorescent protein gene (GFP) tag were used to examine the subcellular localization of the FgABCC9 protein. Hyphae were used to detect GFP fluorescence. The optical microscopic and fluorescence microscopic assays were carried out as shown in Reference [31] by using a Nikon-80i fluorescence microscope (Nikon, Tokyo, Japan).

### 4.9. Statistical Analysis

Student’s *t*-test (implemented in DPS version 12.01 software, [51]) was used to test the significance of differences in mycelial growth, SA content, gene expression, DON production, and the level of disease.

## Figures and Tables

**Table 1:** Primers used in this study.

Primer	Sequence (5′–3′)	Source
LB-F	GCGGGCCCAGGCTACTATGGCTGTT	This study
LB-R	GCGAGCTCAGATGCGAAAGGGTC	This study
RB-F	GGAAGCTTCAATCCCGTTGTTCTGGT	This study
RB -R	GGACTAGTAGCCTGCTTCGTGTTCC	This study
P1-F	CTTTTCTCTTAGGTTTACCCG	[31]
P2-R	TAATGCAGGAGTCGCATAAG	[31]
P3-F	CCCAAAAAGTGCTCCTTCAA	[31]
P4-R	TGTGCTGCAAGGCGATTAA	[31]
ΔJ-U-F	CCTCGGGCGGTCTGTTT	This study
ΔJ-U-R	TTCGGCGTGGGTATGG	This study
ΔJ-D-F	TCCTCGTTCCTGTCTG	This study
ΔJ-D-R	CTCCGATGATGAGAAGT	This study
C-*FgABCC9*-F	TGCCATGGTATTACCGTACTTCCCTA	This study
C-*FgABCC9*-R	GGACTAGTCGACTGTCTACTCGACTC	This study
CJ*-FgABCC9*-F	AAGAAGGCAAAGAAAGCAAA	This study
CJ-*FgABCC9-*R	GAACAAGGCCAGTGATGAGA	This study
Fg-*GAPDH*-F	TGACTTGACTGTTCGCCTCGAGAA	[24]
Fg-*GAPDH*-R	ATGGAGGAGTTGGTGTTGCCGTTA	[24]
Fg-*β-tubulin*-F	GTTGATCTCCAAGATCCGTG	[24]
Fg-*β-tubulin*-R	CATGCAAATGTCGTAGAGGG	[24]
Fg-*elongation Factor1*-F	CCTCCAGGATGTCTACAAGA	[24]
Fg-*elongation Factor1*-R	CTCAACGGACTTGACTTCAG	[24]
*Aox*-F	GACTTGTCATGGTAGATGCCTG	[24]
*Aox*-R	CAGGACGAGCATAACCATTCTC	[24]
w-*GAPDH*-F	AACTGTTCATGCCATCACTGCCAC	[24]
w-*GAPDH*-R	AGGACATACCAGTGAGCTTGCCAT	[24]
*hn-RNP-Q*-F	TCACCTTCGCCAAGCTCAGAACTA	[24]
*hn-RNP-Q*-R	AGTTGAACTTGCCCGAAACATGCC	[24]
Rj-*FgABCC9*-F	GCGGGACCACAAGGGATT	This study
Rj-*FgABCC9*-R	GATGTAGGCACCGTAGACAGACC	This study
*ACT2-F*	CTTGCACCAAGCAGCATGAA	[42]
*ACT2-R*	CCGATCCAGACACTGTACTTCCTT	[42]
*UBQ10-F*	GGCCTTGTATAATCCCTGATGAATAAG	[42]
*UBQ10-R*	AAAGAGATAACAGGAACGGAAACATAGT	[42]
*EF-1α-F*	TGAGCACGCTCTTCTTGCTTTCA	[42]
*EF-1α-R*	GGTGGTGGCATCCATCTTGTTACA	[42]
EDS1-F	GGATAGAAGATGAATACAAGCC	[43]
*EDS1-R*	ACCTAAGGTTCAGGTATCTGT	[43]
*PR1F*	TGGCTATTCTCGATTTTTAATCG	[29]
*PR1R*	CCATTGCACGTGTTCGCAG	[29]
*NPR1-F*	CATTCTCTCAAAGGCCGACT	[44]
*NPR1-R*	AAGACGTTGAGCAAGTGCAA	[44]
*PAD4-F*	ATGGACGATTGTCGATTCGAG	[43]
*PAD4-R*	CTAAGTCTCCATTGCGTCACT	[43]

The underlined are cutting sites for restriction enzymes.
